# Current Recommendations for Nutritional Management of Overweight and Obesity in Children and Adolescents: A Structured Framework

**DOI:** 10.3390/nu11020362

**Published:** 2019-02-09

**Authors:** Shawna Pfeifflé, Fabien Pellegrino, Maaike Kruseman, Claire Pijollet, Magali Volery, Ludivine Soguel, Sophie Bucher Della Torre

**Affiliations:** 1 Department of Nutrition and Dietetics, School of Health Sciences Geneva, University of Applied Sciences and Arts Western Switzerland (HES-SO), Rue des Caroubiers 25, 1227 Carouge, Switzerland; shawna.pfeiffle@gmail.com (S.P.); fabienpellegrino4@gmail.com (F.P.); maaike.kruseman@hesge.ch (M.K.); claire.pijollet@hesge.ch (C.P.); ludivine.soguel@hesge.ch (L.S.); 2 CCNP—Centre de Consultations Nutrition et Psychothérapie, Rue du Vieux-Marché 8, 1207 Genève, Switzerland; magali.volery@ccnp-ge.ch

**Keywords:** pediatric obesity, evidence-based practice, nutritional management, structured framework

## Abstract

Nutritional management is an important component of the treatment of pediatric overweight and obesity, but clinicians struggle to keep abreast with the abundant literature. Therefore, our aim is to provide a tool that integrates the current recommendations and clinical expertise to assist dietitians and other practitioners in their decision making about the nutritional management of pediatric overweight and obesity. To construct this practice-based evidence-informed framework, we conducted a systematic review of the guidelines on nutritional management of pediatric overweight or obesity in 2 databases and in the grey literature. We analyzed and synthesized recommendations of 17 guidelines. We selected the recommendations that were common to at least 30% of the guidelines and added by consensus the recommendations relevant to clinical expertise. Finally, we structured the framework according to the Nutritional Care Process in collaboration with a specialized team of dietitians who assessed its validity in clinical practice. The framework contributes to facilitate the integration of evidence-based practice for dietitians by synthesizing the current evidence, supporting clinical expertise, and promoting structured care following Nutrition Care Process model for children and adolescents with obesity.

## 1. Introduction

Obesity in children and adolescents reaches alarming rates and the World Obesity Federation and the World Health Organization are calling to action in order to reduce the prevalence of pediatric obesity and its many associated physical, psychological, and social consequences [1]. Risk factors for obesity in children and adolescents are numerous and interact with a high level of complexity. Therefore, treatments integrating multiple targets at various levels are deemed the most effective for weight management: i.e., lifestyle interventions including diet, physical activity and behavior changes, and, for patients under 12 years old, targeting the whole family [2,3,4,5,6,7]. Qualified dietitians can contribute effectively to this complex task by providing their expertise on nutritional assessment, diagnosis, intervention and evaluation [8]. In adults, a meta-analysis including 69 studies concluded that dietitian-delivered lifestyle interventions achieve greater relative weight loss, compared to non-dietitian-lead interventions [9].

To provide effective care, dietitians adhere to evidence-based practice (EBP) that is about, according to the International Confederation of Dietetic Associations, “asking questions, systematically finding research evidence, and assessing the validity, applicability and importance of that evidence. This evidence-based information is then combined with the dietitian’s expertise and judgment and the client’s or community’s unique values and circumstances to guide decision-making in dietetics” [10]. Regarding research evidence in obesity treatment, the scientific societies, professional associations and expert groups have issued practice guidelines. These are aimed to “offer coherently sequenced recommendations based on the best available evidence aimed at everyday decision-making situations” [11] and should be “informed by a systematic review of evidence and an assessment of the benefits and harm of alternative care options“ [12]. However, the highest level of evidence (randomized controlled trial) is rarely available in the field of pediatrics, and even less so in dietetics and nutrition where multifaceted interventions are difficult (if not unethical) to standardize in a controlled fashion [13]. In such circumstances, professional expertise is more than crucial in everyday decision-making situations.

With the number of clinical guidelines expanding rapidly, practitioners struggle to stay abreast. Time is often cited as a major barrier for integrating research into practice [14,15] and synthesizing guidelines into a practical tool supporting the clinicians’ decision-making during their interaction with their patients would be helpful. Furthermore, the importance of adapting knowledge to the context is now recognized to support evidence implementation and has been described as an essential step in knowledge translation [16,17,18].

With a focus on EBP, the Academy of Nutrition and Dietetic has developed the Nutritional Care Process (NCP), a systematic approach contributing to high quality nutrition care. This approach helps dietitians structuring their actions in four distinct but interrelated steps: nutrition assessment, diagnosis, intervention and monitoring/evaluation [19,20]. In order to assist the dietitians, but also the other practitioners involved in pediatric weight management to apply these principles, we propose a practice-based evidence-informed framework modeled on the NCP. The aim of this paper is to describe the development of this tool and in particular to: (1) provide a review of the evidence on the dietary and nutritional management of pediatric overweight and obesity, (2) synthesize these evidences into recommendations fitting the NCP-model and (3) propose a framework, drawn from the literature, adapted and expended with professional expertise, for the nutritional management of pediatric obesity. This tool leaves room for individual adaptations based on the professionals’ expertise and the patient’s unique circumstances.

## 2. Materials and Methods

### 2.1. Evidence Selection, Extraction and Analysis

We conducted a systematic literature review of the literature reviews and guidelines on nutritional management of pediatric overweight or obesity in two databases commonly used in healthcare (PubMed and Cochrane Library). In order to improve exhaustiveness, and specifically to avoid missing guidelines emanating from expert groups that have not been published in peer reviewed journals, grey literature was also included. The research equations are detailed in Table 1. The same inclusion and exclusion criteria were applied for reviews and guidelines. Inclusion criteria were: pediatric population (<18 years old), medical guidelines including nutritional management of overweight and obesity, reviews describing a nutritional intervention or treatment, publication date ranging between 2007 and 2017, in French, English or Italian. Exclusion criteria were: papers describing only nutritional prevention or health promotion interventions, focusing on specific ethnicity or gender, with no focus on practical aspects of nutritional intervention, or treating exclusively severe obesity. Two members of the team independently selected the reviews and guidelines, first on the adequacy of the title, then on the abstract and finally on the full-text. At each step, any disagreement was discussed until consensus was reached and a third author was consulted in case of doubt.

An evaluation of the methodological quality of the guidelines was conducted using a simplified grid inspired by the AGREE tool [21] assessing 5 domains (scope and purpose, rigor of development, clarity of presentation, applicability, editorial independence).

Two members of the team independently extracted the recommendations regarding the nutritional management of pediatric overweight and obesity from the 17 included guidelines. The recommendations were isolated, categorized by theme and organized following the 4 steps of Nutritional Care Process (NCP).

### 2.2. Development of the Structured Framework

In a first step, recommendations that were present in at least 30% of the guidelines were used to construct a preliminary framework. Because we could not find previous work publishing on the cut-off point, we chose 30% based on the descriptive analysis of our data. This allowed a selection of a large panel of recommendations. In a second step, the members (*n* = 9) of a specialized group for the prevention and treatment of pediatric obesity recognized by the Swiss Association of Registered Dietitians reviewed the preliminary framework, as well as the table listing all individual recommendations emerging from the guidelines. Based on their clinical expertise, recommendations that are regularly used in clinical practice were identified among the individual recommendations from the guidelines. Even if those recommendations were not mentioned in at least 30% of the guidelines, they were included in the framework while distinctly labeled.

### 2.3. Usability Evaluation

For this third step, in order to verify if the guideline was usable, it was tested by a dietician who was chosen because of her high expertise in the clinical field, her familiarity with the process of guidelines creation and her willingness to participate in the project. This experienced registered dietitian, who did not participate in the second step, selected four cases of children with obesity treated in her practice for an anonymous retrospective case study. Each treatment plan including several consultations was broken down into isolated steps. Nutrition assessment and interventions performed by the dietitian were identified from the patients’ records and compared with the framework. Finally, a semi-structured qualitative interview based on thirteen questions (detailed in Table S1) was conducted with the dietitian in order to assess the suitability of the framework into clinical practice. 

## 3. Results

### 3.1. Evidence Selection

The initial search on PubMed resulted in 115 articles and 4 articles were included after selection. The search on Cochrane identified 3 reviews that did not meet the inclusion criteria. Finally, 16 guidelines were found in the grey literature, of which 3 had been identified through PubMed.

Ultimately, no reviews met the inclusion criteria and we included 17 guidelines, issued by 14 different scientific societies (in alphabetical order: Academy of Nutrition and Dietetics (*n* = 3 in 2007, 2013 and 2015) [22,23,24], American Academy of Pediatrics [8], Canadian Medical Association [25], Endocrine Society [26], European Association for the Study of Obesity [27], Haute Autorité de Santé [28], Institute for Clinical Systems Improvement [29], Institute for Healthy Childhood Weight [2], Institut National d’Excellence en Santé et Services sociaux [30], National Health and Medical Research Council [31], National Institute for Health and Care Excellence [32], New Zealand Ministry of Health (*n* = 2 in 2009 and 2016) [33,34], Scottish Intercollegiate Guidelines Network [35], Società Italiana di Pediatria and Società Italiana di Endocrinologia e Diabetologia Pediatrica [36].

### 3.2. Guidelines Extraction and Analysis

Most guidelines had a good methodological quality. Shortcomings related to the presentation of the methodology used to search and select the evidence. The detail of the evaluation is presented in Supplementary Table S2.

In total, 344 recommendations were extracted, from which 36 (10%) were present in at least half of the guidelines, and 57 (17%) in at least 30% of the guidelines. A summary of the recommendations classified by theme is presented in Table 2. The detail of these recommendations is presented in Supplementary Table S3.

The items related to general recommendations of treatment were similar across all guidelines, while precise recommendations were less common. The levels of evidence reported in the guidelines were mostly low and the recommendations were often based on expert consensus. A comparison of the different levels of evidence across guidelines was impossible because each guideline had a different grading scale. All guidelines considered the treatment of both pediatric overweight and obesity.

### 3.3. Development of the Structured Framework

**Figure 1:**
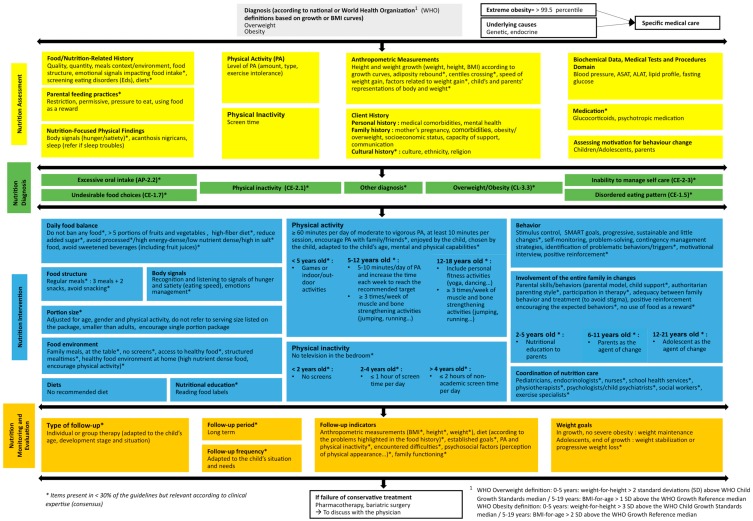
Structured framework on the nutritional management of overweight and obesity in children and adolescents.

The final framework (Figure 1) includes a total of 145 recommendations out of 344 identified in the 17 included guidelines. In the first step of development, we identified 57 recommendations common to at least 30% of the analyzed guidelines. In a second step, based on their clinical expertise, a group of specialized dietitians identified 88 additional recommendations from the guidelines that they regularly used in their practice but that were present in less than 30% of the guidelines. Those 88 recommendations were added to the framework and distinctly labeled. The framework follows the NCP 4-steps structure. We synthesized recommendations and included age groups when possible in the framework. Given that no guideline proposed nutrition diagnoses, we included six of them (in collaboration with the team of specialized dietitians) from the International Dietetics and Nutrition Terminology (IDNT) [37].

### 3.4. Usability Evaluation

The retrospective analysis of four cases revealed that the framework was complete, usable and coherent with clinical practice. Each nutrition intervention retrieved from the 4 patients’ records was already present in the framework and no new recommendation had to be added. The interviewed dietitian highlighted that the framework was helpful to structure the dietetic treatment while leaving enough liberty to adjust the treatment modality to the patient. She used the framework as a checklist to make sure that she did not miss any component. She emphasized that clinical expertise was essential to apply the framework effectively. Also, the framework was perceived as a communication tool that highlights the evidence supporting her practice to external stakeholders.

## 4. Discussion

Dietitians have an essential role in the treatment of obesity among children and adolescents. In this project, we identified the most common recommendations for nutritional management of pediatric overweight and obesity from guidelines and clinical expertise and organized them according to the NCP-model in order to propose a framework assisting expert decision-making.

Our analysis of current guidelines revealed a commonality of many general principles for the dietary and nutritional management of pediatric overweight and obesity, but also a lack of research on specific detailed questions related to everyday practice, such as age-based nutritional intervention or follow-up. While more research on nutrition interventions is necessary, it has to be recognized that this is also very challenging, because food-based or public health interventions involve too many components interacting in complex pathways [4,13]. Dietitians also often collaborate with other health care professionals in multidisciplinary, multi-model interventions. It is therefore almost impossible to isolate the impact of the sole nutritional care [39]. Pragmatic trials are an example of a new trend toward studies designed to inform practice and show real-world effectiveness of interventions [40]. Dietitians are uniquely and favorably positioned to participate in research projects using feasible and appropriate research designs to answer specific and practical questions.

Most of the included guidelines targeted medical treatment of pediatric overweight and obesity, resulting in few details of the nutritional management. Only 17% of the recommendations were commonly to 30% or more of the guidelines. The expertise of clinical dietitians seems essential to apply the framework with specific, pragmatic, and detailed advice. Since no guideline indicated durations of the first or following visits, the dietitians will have to adapt the content to their time constraints and the individual possibilities of their patients. In fact, the adaptation of knowledge to local context, or to dietetic practice in this case, is part of the steps of knowledge transfer process [18,41]. Of course, this step might represent a limitation of the tool as it introduces non-evidence-based recommendations, even if those are clearly labeled as such. This tool does not claim to be definitive. Indeed, it will have to be modified and enhanced after a period of field-testing that will enlarge the preliminary usability evaluation carried out by only one specialized dietitian. We also acknowledge that fact that we did not assess the methodological quality of the guidelines included in our analysis; therefore, recommendations from guidelines of variable methodological quality have the same weight in the framework. Lastly, the selection of guidelines was limited to the three languages mastered by our team what could lead to a selection bias.

Nevertheless, the strength of this paper is to provide a framework synthesizing the current literature based on a systematic analysis of each guideline, the extraction of each single recommendation and the expertise from clinical professionals. Our framework is coherent with the NCP and reinforces the use of this structured four-steps process to promote high quality of dietetics and nutrition care. Finally, the retrospective validity assessment demonstrated the relationship between the framework and the clinical practice and suggested a high potential of use by clinical dietitians.

## 5. Conclusions

This paper synthesizes the current recommendations on nutritional management of pediatric overweight and obesity with the expertise from experienced dietitians in an attempt to create a tool aiming to facilitate the integration of EBP for dietitians and to promote structured care following NCP-model.

## Figures and Tables

**Table 1:** Databases and research equations for the systematic review of guidelines on nutritional management of pediatric overweight or obesity.

Database	Search Terms or Search Equation
Medline via PubMed	(terms in all fields): ((pediatric AND obesity) OR (pediatric obesity) OR (obesity AND child)) AND (guideline OR algorithms OR evidence-based medicine) AND (diet therapy OR nutrition therapy OR case management OR patient care management OR dietetics/education); filters: 10 years, Humans, Child: birth-18 years.
The Cochrane Library	pediatric obesity, pediatric obesity guidelines and treatment, obesity.
Grey literature research (Google, Bing)	guidelines, pediatric/childhood obesity, nutritional management, algorithm, the name of the country (Australia, Canada, Scotland, United States, France, Italy, New-Zealand, United Kingdom), the name of the scientific society (Academy of Nutrition and Dietetics, American Academy of Pediatrics, Canadian Medical Association, European Association for the Study of Obesity, Haute Autorité de Santé, Institute for Clinical Systems Improvement, Institute for Healthy Childhood Weight, Institut National d’Excellence en Santé et Services sociaux, National Health and Medical Research Council, National Institute for Health and Care Excellence, New Zealand Ministry of Health, Scottish Intercollegiate Guidelines Network, Società Italiana di Pediatrica, The Endocrine Society).

**Table 2:** Recommendations regarding nutritional assessment, diagnosis, intervention and monitoring and evaluation, frequency of citation and references of the 17 included guidelines.

Theme	Recommendation	*N* of Citation	Reference
**Nutritional Assessment**			
Food/Nutrition-related history	Assess eating patterns	13	[8,22,23,24,25,26,27,28,29,30,31,32,33,34,35,36]
	Assess food quality and quantity (including portion sizes and overeating)	7	[8,22,23,24,27,28,31,33,34,36]
	Assess the presence of sugar-sweetened beverages and juices	7	[8,22,23,24,25,26,27,31,33,34]
	Assess meals structure and distribution	7	[8,22,23,24,25,27,28,31,33,34]
	Assess meals context and environment	6	[26,27,28,29,33,34,36]
	Assess the presence of snacking	3	[27,28,31]
Anthropometric measurements	Calculate BMI	13	[8,22,23,24,25,26,27,28,29,30,31,32,33,34,35,36]
	Analyze growth curves	11	[8,25,26,27,28,29,30,31,32,33,34,36]
	Assess the factors conducting to a weight gain	2	[30,31]
	Assess the precocity of the adiposity rebound	1	[28]
Biochemical data, medical tests and procedures	Interpret of blood pressure	10	[8,25,26,27,28,29,31,32,33,34,36]
	Interpret of blood lipids	10	[8,25,26,27,28,29,30,32,33,34,36]
	Analyze blood glucose	9	[8,25,26,27,28,29,30,32,36]
	Assess hepatic tests	7	[25,26,27,28,29,32,36]
Nutrition-focused physical findings	Assess the presence of acanthosis nigricans	7	[8,26,27,29,31,33,34,35]
	Assess sleep	6	[8,27,28,30,31,33,34]
	Assess food sensations	1	[28]
Physical activity	Assess physical activity level	12	[8,22,23,24,25,26,28,29,30,31,32,33,34,35,36]
	Assess sedentary behaviors (especially the screen time)	11	[8,22,23,24,25,26,27,28,29,30,31,33,34,35]
Client history	Assess comorbidities	11	[8,25,26,27,29,30,31,32,33,34,35,36]
	Assess family history	12	[8,25,26,27,28,29,30,31,32,33,34,35]
	Assess mental health	8	[8,22,23,24,26,30,31,32,33,34,35]
	Assess social/family environment	8	[22,23,24,25,26,28,30,31,32,35]
	Assess parental feeding practices (restrictive, permissive, pressure to eat, food as reward)	3	[22,23,24,26,28]
Eating Disorders screening	Screen for eating disorders	9	[8,22,23,24,26,28,29,30,31,35,36]
	Assess the presence of Binge Eating Disorders	5	[8,26,28,31,36]
Change motivation	Assess motivation and readiness to change	9	[8,25,27,28,29,30,31,32,33,34,35]
**Nutritional Diagnosis**			
	No guideline referred to nutritional diagnosis.	0	
**Nutritional Intervention**			
Meal structure	Structure eating with 3 meals and 2 snacks per day	5	[2,8,31,35,36]
	Avoid snacking	3	[8,26,36]
Food balance	Promote fruit and vegetable consumption	7	[8,26,27,29,33,34,35,36]
	Balance diet and eating habits	6	[25,28,30,32,33,34,36]
	Avoid high energy density and low nutritional density foods (fast-foods, take-out, sugar sweetened beverages and juices)	5	[2,26,29,33,34,36]
	Do not forbid any food	3	[28,31,35]
	Avoid sugar sweetened beverages and juices	4	[8,26,29,33,34]
	Limit sugar sweetened beverages and juices consumption	3	[27,35,36]
Portion sizes	Follow national recommendations	4	[29,33,34,35,36]
Diets	Avoid any type of restrictive diet	8	[8,22,23,24,25,28,32,33,34,35,36]
	Follow the “stop/traffic light diet” (The traffic light diet divides food groups into 3 categories: green (low-energy, high-nutrient foods, may be eaten often), yellow (moderate-energy foods, may be eaten in moderation), and red (high-energy, low-nutrient foods, should be eaten sparingly). [38]	4	[22,23,24,25,30,36]
Meal environment	Eat as a family	6	[8,27,29,30,31,35]
	Avoid screen during meals	3	[27,31,35]
	Offer healthy foods at home	1	[31]
Food sensations	Work on the recognition and respect of the food sensations	6	[2,28,30,31,35,36]
Family implication	Families should be included in the treatment	14	[2,8,22,23,24,25,26,27,28,29,30,31,32,33,34,35,36]
	Family behavior should not be different than the child with excess weight to avoid stigmatization	8	[2,8,27,28,30,32,33,34,35]
Lifestyle intervention	Multifactorial treatment including the food balance, the physical activity and the behavior management	10	[2,22,23,24,26,28,29,30,31,32,33,34,35]
Physical activity	At least 60 min of moderate to vigorous activity per day (at least 10 min per session)	10	[2,8,27,28,29,30,31,32,35,36]
	Importance of the child’s choice and pleasure in the activity	8	[8,25,27,29,30,32,33,34,35]
Sedentarity	Reduce non-academic screen time to max. 2 h both in week days and weekend days	8	[2,8,26,27,29,30,33,34,35]
Behavior management	Practice *stimulus control*	8	[2,22,23,24,25,29,30,32,33,34,35]
	Use motivational counseling	8	[2,8,25,26,27,28,29,30]
	Set *SMART* objectives with the child and/or the parents	8	[2,8,25,29,30,31,33,34,35]
	Introduce behavior and objectives self-monitoring	7	[2,8,25,29,30,33,34,35]
Care coordination and implication	Dietitian	7	[8,22,23,24,25,26,27,28,30]
	Pediatrician	6	[8,25,27,28,29,30]
	Other professionals		Details in Table S1
	Interdisciplinary teams	3	[24,25,30]
Pharmacotherapy	Recommend to use *Orlistat* in specific conditions but warn about its low efficacy	11	[8,22,23,24,25,26,29,30,31,32,33,34,35,36]
Bariatric surgery	In general, not recommended. Indications and contraindications were different between guidelines.		Details in Table S1
Comorbidities	Consider comorbidities during the intervention	9	[8,26,27,28,29,30,32,35,36]
**Nutritional Monitoring and evaluation**			
Follow-up	Individual or group follow-up depending on the child’s situation	2	[22,23,24,36]
	Long-term follow-up	4	[2,25,31,32]
Monitoring indicators	Monitor anthropometric measurements	10	[2,8,22,23,24,26,28,29,31,33,34,35,36]
	Monitor food balance and physical activity	4	[8,29,31,36]
	Monitor changes according to the objectives	1	[28]
Weight management	Weight stabilization for growing children	7	[22,23,24,26,27,28,29,31,32]

## Supplementary Materials

The following are available online at https://www.mdpi.com/2072-6643/11/2/362/s1, Table S1: Semi-structured questionnaire evaluating the usability of the framework in practice, Table S2: Quality assessment of guidelines included in the framework, Table S3: Detailed recommendations regarding nutritional assessment, diagnosis, intervention and monitoring and evaluation, frequency of citation and references of the guidelines.
