# Supplementary material for: Current Recommendations for Nutritional Management of Overweight and Obesity in Children and Adolescents: A Structured Framework
Source: Nutrients. 2019 Feb 9;11(2):362. doi: 10.3390/nu11020362 (PMC6412470; doi:10.3390/nu11020362)
Supplement: Supplementary file 1 [file nutrients-11-00362-s001.zip › Supplementary_material_R3/Table_S1_Usability_Evaluation_R2_V1.docx]

Online Supplementary Material

Current recommendations for nutritional management of overweight and obesity

in children and adolescents: A structured framework

**Table S1.** Semi-structured questionnaire evaluating the usability of the framework in practice.

| Usability of the framework | 1. What does the framework add to the process of nutritional care? 2. Would the use of the framework have changed your decisions? 3. What specific items of the framework bring new element to your practice ? 4. When would you have used the framework – before, during or after the session? 5. How could the framework help you integrating the Nutrition Care Process in your practice? |
| --- | --- |
| Content of the framework | 1. Is there differences between your knowledge and the items of the framework? 2. Do some of the items suprized you ? Why ? 3. Do the nutritional diagnosis presented in the framework fit with your practice? 4. From your point of view, is there any nutritional diagnosis missing? 5. Do age groups presented in the framework match your practice? 6. Would you have any suggestion to add items or modify the framework? |
| Format of the framework | 1. Is the framework readable and clear? 2. Is the format practical for use in professional situations? |
