# Supplementary material for: Current Recommendations for Nutritional Management of Overweight and Obesity in Children and Adolescents: A Structured Framework
Source: Nutrients. 2019 Feb 9;11(2):362. doi: 10.3390/nu11020362 (PMC6412470; doi:10.3390/nu11020362)
Supplement: Supplementary file 1 [file nutrients-11-00362-s001.zip › Supplementary_material_R3/Table_S2_Quality_R2_V1.docx]

Online Supplementary Material

Current recommendations for nutritional management of overweight and obesity

in children and adolescents: A structured framework

**Table S2:** Quality assessment of guidelines included in the framework, using a simplified grid inspired by the AGREE tool^1^. *For more readability, all guidelines are indicated with their abbreviation in the table. Full description and references can be found at the end of the document.*

|  |  | AAP | AND 2007 | AND 2013 | AND 2015 | CMA | EASO | EnS | HAS | ICSI | IHCW | INESSS | NHMRC | NICE | NZMH 2009 | NZMH 2016 | SIGH | SIP |
| --- | --- | --- | --- | --- | --- | --- | --- | --- | --- | --- | --- | --- | --- | --- | --- | --- | --- | --- |
| Scope and purpose | 1. The overall objective(s), health question(s) covered by the guideline and the targeted population are specifically described. | Y | Y | Y | Y | UC | No information on methodology | Y | No information on methodology | UC | UC | Y | Y | Y | Y | Y | Y | UC |
| Rigor of development | 1. Systematic methods were used to search and select the evidence. | N | Y | Y | Y | UC |  | UC |  | UC | UC | Y | Y | Y | Y | Y | UC | UC |
| Clarity of presentation | 1. The results clearly presented. | Y | Y | Y | Y | Y |  | Y |  | Y | Y | Y | Y | Y | Y | Y | Y | Y |
| Applicability | 1. The results can be implemented in practice. | Y | Y | Y | Y | Y |  | Y |  | Y | Y | Y | Y | Y | Y | Y | Y | Y |
| Editorial independence | 1. The views of the funding body or sponsorship have not influenced the content of the guideline | Y | Y | Y | Y | Y |  | Y |  | Y | Y | Y | Y | Y | Y | Y | Y | Y |

Y = Yes, N = No, UC = Unclear, NA = Not applicable

**Full description and references of the 17 guidelines:**

AAP American Academy of Pediatrics ^2^

AND Academy of Nutrition and Dietetics ^3-5^

CMA Canadian Medical Association ^6^

EASO European Association for the Study of Obesity ^7^

EnS Endocrine Society ^8^

HAS Haute Autorité de Santé ^9^

ICSI Institute for Clinical Systems Improvement ^10^

IHCW Institute for Healthy Childhood Weight ^11^

INESSS Institut national d’excellence en santé et en service sociaux ^12^

NHMRC National Health and Medical Research Council ^13^

NICE National Institute for Health and Care Excellence ^14^

NZMH New Zealand Ministry of Health ^15,16^

SIGN Scottish Intercollegiate Guidelines Network ^17^

SIP Società Italiana di Pediatria ^18^

**References**

1. Brouwers M, Kho M, Browman G, et al. AGREE II: Advancing guideline development, reporting and evaluation in healthcare. *CMAJ.* 2010;182:E839-842.

2. Spear BA, Barlow SE, Ervin C, et al. Recommendations for Treatment of Child and Adolescent Overweight and Obesity. *Pediatrics.* 2007;120(Supplement 4):S254-S288.

3. Academy of Nutrition and Dietetics. Pediatric Weight Management. Major recommendations (2007). 2007; <https://www.andeal.org/vault/pq57.pdf>. Accessed 10.12.2018.

4. Academy of Nutrition and Dietetics. Pediatric Weight Management. Major recommendations (2015). 2015; <https://www.andeal.org/vault/pq140.pdf>. Accessed 10.12.2018.

5. Hoelscher DM, Kirk S, Ritchie L, Cunningham-Sabo L. Position of the Academy of Nutrition and Dietetics: Interventions for the Prevention and Treatment of Pediatric Overweight and Obesity. *Journal of the Academy of Nutrition and Dietetics.* 2013;113(10):1375-1394.

6. Lau DC, Douketis JD, Morrison KM, et al. 2006 Canadian clinical practice guidelines on the management and prevention of obesity in adults and children [summary]. *CMAJ.* 2007;176(8):S1-13.

7. Baker JL, Farpour-Lambert NJ, Nowicka P, Pietrobelli A, Weiss R, Childhood Obesity Task Force of the European Association for the Study of Obesity. Evaluation of the overweight/obese child--practical tips for the primary health care provider: recommendations from the Childhood Obesity Task Force of the European Association for the Study of Obesity. *OFA.* 2010;3(2):131-137.

8. Styne DM, Arslanian SA, Connor EL, et al. Pediatric Obesity-Assessment, Treatment, and Prevention: An Endocrine Society Clinical Practice Guideline. *J Clin Endocrinol Metab.* 2017;102(3):709-757.

9. Haute Autorité de Santé. Surpoids et obésité de l'enfant et de l'adolescent. 2011; <https://www.has-sante.fr/portail/jcms/c_964941/fr/surpoids-et-obesite-de-l-enfant-et-de-l-adolescent-actualisation-des-recommandations-2003>. Accessed 22.12.2018.

10. Institute for Clinical Systems Improvement. *Prevention and Management of Obesity for Children and Adolescents.* 2013.

11. Altman M, Wilfley DE. Evidence update on the treatment of overweight and obesity in children and adolescents. *J Clin Child Adolesc Psychol.* 2015;44(4):521-537.

12. Institut national d'excellence en santé et en services sociaux. *Traitement de l'obésité des enfants et des adolescents en 1re et 2e ligne: guide de pratique clinique. Volet I.* Montreal, Québec: INESSS;2012.

13. National Health Medical Research Council. *Clinical practice guidelines for the management of overweight and obesity in adults, adolescents and children in Australia.* Melbourne: National Health and Medical Research Council;2013.

14. National Clinical Guideline Centre. *Obesity: Identification, Assessment and Management of Overweight and Obesity in Children, Young People and Adults: Partial Update of CG43.* London: National Institute for Health and Care Excellence (UK); 2014.

15. Ministry of Health. *Clinical Guidelines for Weight Mangagement in New Zealand Children and Young People.* Wellington: Ministry of Health;2016.

16. Ministry of Health, Clinical Trials Research Unit. *Clinical Guidelines for Weight Management in New Zealand Children and Young People.* Wellington: Ministry of Health;2009.

17. Scottish Intercollegiate Guidelines Network. *Management of obesity: a national clinical guideline.* Edinburgh: Scottish Intercollegiate Guidelines Network; 2010.

18. Società Italiana di Pediatria, Società Italiana di Endocrinologia e Diabetologia Pediatrica. Consensus su diagnosi, trattamento e prevenzione dell'obesita del'bambino e dell'adolescente. 2017; <https://docs.sip.it/Consensus_Obesita_2017.pdf>. Accessed 22.12.2018.
