# Supplementary material for: Current Recommendations for Nutritional Management of Overweight and Obesity in Children and Adolescents: A Structured Framework
Source: Nutrients. 2019 Feb 9;11(2):362. doi: 10.3390/nu11020362 (PMC6412470; doi:10.3390/nu11020362)
Supplement: Supplementary file 1 [file nutrients-11-00362-s001.zip › Supplementary_material_R3/Table_S3_Detailled_Recommendations_R2_V1.docx]

Online Supplementary Material

Current recommendations for nutritional management of overweight and obesity

in children and adolescents: A structured framework

**Table S3.** Detailed recommendations regarding nutritional assessment, diagnosis, intervention and monitoring and evaluation, frequency of citation and references of the guidelines. *For more readability, all guidelines are indicated with their abbreviation in the table. Full description and references can be found at the end of the document*

ASSESSEMENT

| Recommendations mentioned in guidelines | Number of citation | References of the guidelines |
| --- | --- | --- |
| Recommendations regarding assessment of eating habits | | |
| Eating habits | 13 | AAP, AND, CMA, EASO, EnS, HAS, ICSI, INESSS, NHMRC, NICE, NZMH, SIGN, SIP |
| Food quality (for example: saturated fatty acids, added sugars, salt, high energy density food, industrial products, prepared food, fast-food…) | 7 | AAP, AND, EASO, HAS, NHMRC, NZMH, SIP |
| Food structure (especially the presence of breakfast) | 7 | AAP, AND, CMA, EASO, HAS, NHMRC, NZMH |
| Sugar-sweetened beverages (including juices) consumption | 7 | AAP, AND, CMA, EASO, EnS, NHMRC, NZMH |
| Frequency and number of food intakes | 6 | AAP, AND, CMA, EASO, HAS, SIP |
| Meal context and environment | 6 | EASO, EnS, HAS, ICSI, NZMH, SIP |
| Fruits and vegetables consumption | 6 | AAP, AND, CMA, EASO, NHMRC, NZMH |
| Frequency and quality of snacks | 5 | AAP, AND, EASO, EnS, NZMH |
| Portion size | 4 | AAP, AND, EASO, SIP |
| Meals taken outside (fast-foods, take-away, restaurants…) | 4 | AAP, AND, EnS, NHMRC |
| Snacking | 3 | EASO, HAS, NHMRC |
| Food at school | 2 | AAP, EASO |
| Emotional signals impacting food intake (boredom, stress, loneliness…) | 2 | EnS, HAS |
| Meal place and schedule | 1 | EASO |
| Alcohol consumption | 1 | NZMH |
| Help with the meal preparation | 1 | NZMH |
| Dairy products consumption | 1 | AND |
| Relation to food | 1 | INESSS |
| Preferences, aversions | 1 | SIP |
| Use of condiments | 1 | SIP |
| Cooking techniques | 1 | SIP |
| Dishes presentation | 1 | SIP |
| Dieting | 1 | NHMRC |
| Recommendations regarding assessment of anthropometric data | | |
| Considering both overweight and obesity in nutritional care | 14 | AAP, AND, CMA, EASO, EnS, HAS, ICSI, IHCW, INESSS, NHMRC, NICE, NZMH, SIGN, SIP |
| BMI (with BMI curves) | 13 | AAP, AND, CMA, EASO, EnS, HAS, ICSI, INESSS, NHMRC, NICE, NZMH, SIGN, SIP |
| Height and weight growth and its speed (with growth curves) | 11 | AAP, CMA, EASO, EnS, HAS, ICSI, INESSS, NHMRC, NICE, NZMH, SIP |
| Child’s and parents representations of body and weight | 4 | EnS, HAS, ICSI, NICE |
| Tanner stages | 3 | AAP, NICE, NZMH |
| Factors related to weight gain | 2 | INESSS, NHMRC |
| Centiles crossing | 2 | HAS, NHMRC |
| Waist-to-height ratio | 2 | HAS, NHMRC |
| Waist size | 2 | AND, EASO |
| Early adiposity rebound | 1 | HAS |
| BMI z-score | 1 | AND |
| Weight-to-height ratio with children under 5 years | 1 | NZMH |
| Recommendations regarding assessment of clinical parameters | | |
| Blood pressure | 10 | AAP, CMA, EASO, EnS, HAS, ICSI, NHMRC, NICE, NZMH, SIP |
| Lipid profile (triglycerides, HDL-cholesterol, LDL-cholesterol, total cholesterol, cholesterol-HDL cholesterol ratio) | 10 | AAP, CMA, EASO, EnS, HAS, ICSI, INESSS, NICE, NZMH, SIP |
| Fasting glucose | 9 | AAP, CMA, EASO, EnS, HAS, ICSI, INESSS, NICE, SIP |
| Liver profile (ASAT, ALAT) | 7 | CMA, EASO, EnS, HAS, ICSI, NICE, SIP |
| Presence of a secondary etiology of obesity (genetic, endocrine) | 5 | AAP, NHMRC, NICE, SIGN, SIP |
| HbA1C (glycated hemoglobin) | 4 | EnS, HAS, NICE, NZMH |
| Determine energy expenditure with indirect calorimetry or prediction equation (2005 US Institute of Medicine) | 1 | AND |
| Micronutrient deficiencies (with a blood test) | 1 | EASO |
| Sleep-quality test (pulse oximetry) | 1 | NZMH |
| Recommendations regarding nutritional clinical assessment | | |
| Presence of acanthosis nigricans | 7 | AAP, EASO, EnS, ICSI, NHMRC, NZMH, SIGN |
| Quality of sleep | 6 | AAP, EASO, HAS, INESSS, NHMRC, NZMH |
| Sweating and discomfort in clothes | 1 | SIGN |
| Presence of edema | 1 | EnS |
| Oral health | 1 | NHMRC |
| Food sensations | 1 | HAS |
| Recommendations regarding assessment of physical activity and sedentary behaviors | | |
| Level of physical activity (length, frequency) | 12 | AAP, AND, CMA, EnS, HAS, ICSI, INESSS, NHMRC, NICE, NZMH, SIGN, SIP |
| Inactivity (screen time) | 11 | AAP, AND, CMA, EASO, EnS, HAS, ICSI, INESSS, NHMRC, NZMH, SIGN |
| Practice of a sport | 3 | AND, EnS, NZMH |
| Intolerance to physical activity | 3 | HAS, NHMRC, SIGN |
| Factors related to physical activity: environment, social support and obstacles | 1 | AAP |
| Recommendations regarding assessment of medical conditions and prior treatments | | |
| Comorbidities (for example: gastroesophageal reflux, non-alcoholic steatohepatitis, joint problems, snoring, respiratory difficulties, obstructive sleep apnea syndrome, abdominal pain, glucose intolerance, diabetes, insulin resistance, dyslipidemia, high blood pressure, eating disorder, stigmatization, asthma, polykistic ovary syndrome…) | 11 | AAP, CMA, EASO, EnS, INESSS, ICSI, NHMRC, NICE, NZMH, SIGN, SIP |
| Family history (for example: comorbidities, anthropometric measurements, corpulence, history of obesity or cardiovascular disease…) | 11 | AAP, CMA, EASO, EnS, HAS, ICSI, INESSS, NHMRC, NICE, NZMH, SIGN |
| Mental health (for example: depression, anxiety, stigmatization, self-esteem, victimization, isolation, mental retardation, body dissatisfaction…) | 8 | AAP, AND, EnS, INESSS, NHMRC, NICE, NZMH, SIGN |
| Family and/or social environment (for example: support capability, organization, difficulties, socio-economic status, school functioning, communication) | 8 | AND, CMA, EnS, HAS, INESSS, NHMRC, NICE, SIGN |
| Pregnancy (for example: gestational diabetes, high blood pressure, birth weight, intrauterine growth retardation, smoking, BMI…) | 5 | CMA, EASO, HAS, NHMRC, NZMH |
| Treatments (for example: glucocorticoids, psychotropic) | 4 | AND, EASO, NICE, NZMH |
| Parental feeding practices (for example: restrictive, permissive, pressure to eat, food as reward) | 3 | AND, EnS, HAS |
| Menstrual cycle | 3 | CMA, EASO, NHMRC |
| Culture, values, ethnicity and religion | 2 | AAP, AND |
| Geographic environment (parks/gardens, shops, neighborhood) | 1 | AAP |
| Recommendations regarding eating disorder screening and assessment | | |
| Eating disorder screening | 9 | AAP, AND, EnS, HAS, ICSI, INESSS, NHMRC, SIGN, SIP |
| Presence of binge-eating disorder | 5 | AAP, EnS, HAS, NHMRC, SIP |
| Presence of compensatory behaviors | 2 | AAP, EnS |
| Eating disorder screening with SCOFF survey | 1 | ICSI |
| Presence of diet’s loss of control | 1 | SIP |
| Presence of night feeding syndrome | 1 | HAS |
| Presence of prandial overeating | 1 | NHMRC |
| Presence of anorexia nervosa | 1 | EnS |
| Recommendations regarding assessment of motivation to change | | |
| Motivation to change | 9 | AAP, CMA, EASO, HAS, ICSI, INESSS, NHMRC, NICE, NZMH, SIGN |
| Parents and/or family motivation | 4 | AAP, INESSS, NHMRC, NZMH |
| Recommendations regarding age categories in guidelines | | |
| Age categories mentioned | 7 | AAP, AND, HAS, ICSI, INESSS, NZMH, SIP |

INTERVENTION

| Item mentioned in guidelines | Number of guideline-s mentioning it | References of the guidelines |
| --- | --- | --- |
| Recommendations regarding intervention on eating structure | | |
| Food structure with 3 meals and 2 snacks | 5 | AAP, IHCW, NHMRC, SIGN, SIP |
| Regular meals | 3 | EnS, HAS, IHCW |
| Avoid snacking | 3 | AAP, EnS, SIP |
| Create an individualized meal plan | 2 | AND, CMA |
| Do not create a meal plan, whether it is hypocaloric or not. | 1 | SIP |
| Overweight children (1-2 years old):   - 3 meals per day - 2 snacks | 1 | AAP |
| N2: Structured Weight Management :   - Meal plan created by a dietitian - Food structure (3 meals and 1-2 snacks) - No snacking or caloric drinks consumption between meals | 2 | AAP, ICSI |
| N3: Comprehensive multidisciplinary intervention :   - Food control - Meal plan in the short term - Structured physical activity plan | 2 | AAP, ICSI |
| Recommendations regarding intervention on healthy eating | | |
| Promote fruit and vegetable consumption | 7 | AAP, EASO, EnS, ICSI, NZMH, SIGN, SIP |
| Promote healthy and balanced diet | 6 | CMA, HAS, INESSS, NICE, NZMH, SIP |
| Promote low energy-density foods and avoid high energy-density and low nutritional quality (fast-food, take-away, energy/sugar-sweetened beverages and juices) | 5 | EnS, ICSI, IHCW, NZMH, SIP |
| Promote high fiber diet | 4 | EnS, ICSI, NZMH, SIP |
| Promote complex sugars (preferably whole grains) | 4 | EASO, NZMH, SIGN, SIP |
| Eliminate sugar-sweetened beverages and juices | 4 | AAP, EnS, ICSI, NZMH |
| Avoid sugar-sweetened beverages and juices, prefer water | 3 | EASO, SIGN, SIP |
| Consume dairy products preferably low fat | 3 | ICSI, NZMH, SIGN |
| Limit salt | 3 | EnS, NZMH, SIGN |
| Do not forbid any food | 3 | HAS, NHMRC, SIGN |
| Promote low fat food consumption | 3 | EASO, EnS |
| Promote high nutritional quality breakfast | 2 | AAP, ICSI |
| Promote low fat protein food sources (except dairy) | 2 | NZMH, SIGN |
| Reduce added sugars and avoid processed food (saturated fatty acids, sugar, salt) | 2 | EnS, NZMH |
| Define an appropriate caloric target to create a caloric restriction (children from 6 years and teenagers) | 2 | AND, IHCW |
| Eat healthy snacks | 1 | NZMH |
| Eat ≥2 servings of fish per week, including at least 1 serving of fatty fish | 1 | SIGN |
| Individualized approach according to preferences | 1 | NICE |
| Overweight children (1-2 years old) :   - To avoid sugar-sweetened beverages, juices and milk in excess (>4-5-7dl) - Prefer water | 1 | AAP |
| N1: Prevention Plus :   - 5 servings of fruit and vegetables per day - Max. 1 serving of sugar-sweetened beverages per day - Daily healthy breakfast | 2 | AAP, ICSI |
| N2: Structured Weight Management :   - Meal plan created by a dietitian with a balanced repartition of macronutrients and low energy-density food | 1 | AAP |
| N2: Structured Weight Management :   - Healthy snacks | 1 | ICSI |
| Recommendations regarding intervention on portion size | | |
| Limit portion sizes according to national recommendations | 4 | ICSI, NZMH, SIGN, SIP |
| Learn how to control portion sizes | 1 | EnS |
| Promote single portions | 1 | EnS |
| Do not rely on the portions indicated on the package | 1 | ICSI |
| Consume smaller portions than those of the parents | 1 | SIGN |
| Recommendations regarding diet prescription | | |
| No diet can be recommended (no strong evidence) | 8 | AAP, AND, CMA, HAS, NICE, NZMH, SIGN, SIP |
| « Stop/Traffic light diet^[[1]](#footnote-1)^ » may be used | 4 | AND, CMA, INESSS, SIP |
| In case of severe obesity, to use a hypocaloric diet under medical supervision | 3 | AND, HAS, SIP |
| Do not use any hypocaloric diet | 2 | EnS, INESSS |
| Induce a caloric restriction while covering nutritional needs | 1 | EnS |
| Use very hypocaloric diet during a short period and follow-up after in order to maintain weight loss | 1 | NHMRC |
| N4: Tertiary care intervention :   - Very low caloric diet: no evidence on the long term | 1 | AAP |
| Recommendations regarding intervention on food environment | | |
| Eat as a family/Family meals | 6 | AAP, EASO, ICSI, INESSS, NHMRC, SIGN |
| Eat without screen | 3 | EASO, SIGN, NHRMC |
| Establish a regular meal and sleep schedule | 3 | ICSI, IHCW, INESS |
| Create a healthy environment at home (high nutritional quality food and encouragement of physical activity) | 3 | AAP, IHCW, SIGN |
| Limit outside meals | 2 | AAP, ICSI |
| Eat at the table | 1 | EASO |
| Have healthy food available | 1 | NHMRC |
| Overweight children (1-2 years old) :   - At the table - As a family - Without screen | 1 | AAP |
| N1: Prevention Plus :   - At least 5-6 meals per week as a family - To cook at home as much as possible | 1 | AAP |
| Recommendations regarding intervention on food sensations and emotional eating | | |
| Work on the recognition of hunger and satiety signals | 6 | HAS, IHCW, INESSS, NHMRC, SIGN, SIP |
| Work on the emotions management | 1 | NHMRC |
| Work on eating desire and pleasure | 1 | HAS |
| Learn how to manage « food cravings » | 1 | IHCW |
| Recommendations regarding intervention on nutritional education | | |
| Teach how to read nutritional labels | 1 | EnS |
| Nutritional education should be done by a professional, preferably a dietitian | 1 | CMA |
| Take into account the social, economic and cultural contexts for the nutritional education | 1 | HAS |
| Target nutritional education on the lack of knowledge identified during nutritional assessment | 1 | AND |
| N1: Prevention Plus :   - To let the child regulate his meals and avoid restrictive behaviors (< 12 years old) | 1 | AAP |
| Recommendations regarding intervention on therapeutic education | | |
| Explain long-term treatment for overweight/obesity and caution against drastic practices aimed at short-term effects | 1 | EASO |
| Use interview techniques based on cognitive-behavioral therapy for children and parents | 1 | SIP |
| Recommendations regarding intervention on family involvement | | |
| Involve family in the treatment | 14 | AAP, AND, CMA, EASO, HAS, ICSI, IHCW, INESSS, NICE, NHMRC, NZMH, SIGN, SIP |
| Adequacy between family behavior and child’s treatment (to avoid stigmatization) | 8 | AAP, EASO, HAS, IHCW, INESSS, NICE, NZMH, SIGN |
| Develop parenting skills (in terms of food, physical activity and education) | 5 | AAP, EnS, HAS, INESSS, SIGN |
| Parents are in charge of grocery shopping and meal preparation | 4 | AAP, EASO, NICE, NZMH |
| Promote positive reinforcement by the family to encourage healthy behaviors | 4 | AAP, ICSI, IHCW, NZMH |
| Do not use food rewards | 4 | HAS, ICSI, IHCW, SIGN |
| Family involvement during interviews with the child and, according to age and maturity, with the teenager | 2 | INESS, NHMRC |
| Explain to the family the benefits of the changes to maintain their motivation | 2 | EASO, SIGN |
| Children (6-11 years old): family-based treatment with parents as agents of change | 2 | AND, ICSI |
| Teenagers (12-21 years old): family-based treatment with teenager as agent of change | 2 | AND, ICSI |
| Use education methods that increase child or teenager self-esteem and body-esteem | 2 | EnS, IHCW |
| Promote democratic/autocratic parenting style | 2 | HAS, INESSS |
| Parents are information mediators | 1 | NHMRC |
| Children (2-5 years old): nutritional education to the parents; highlight expected behaviors and reduce attention to difficulties; promote process/behavior more than result | 1 | ICSI |
| Teenagers: flexibility of parents in communication | 1 | ICSI |
| Make parents aware of their own eating habits, physical activity, and behaviors | 1 | SIGN |
| Inform parents of the cardiovascular risks associated with childhood obesity | 1 | SIGN |
| Personalize the care according to needs (as a family, apart) | 1 | AND |
| The family bear the main responsibility in the treatment (predominately < 12 years old) | 1 | NICE |
| Overweight children (1-2 years old):   - No restriction - Provide a healthy food rich in fruit and vegetables | 1 | AAP |
| N1: Prevention Plus :   - Involve all the family in lifestyle changes | 1 | AAP |
| N3: Comprehensive multidisciplinary intervention :   - Involve the family for the children aged less than 12 years old, followed by a progressive decrease of implication - Improve home environment | 3 | AAP, AND, ICSI |
| Recommendations regarding implication of other stakeholders | | |
| Involve other environment stakeholders (schools, State) linked with the child (predominately for prevention or research) | 6 | AAP, EnS, IHCW, NICE, SIGN, SIP |
| Recommendations regarding intervention on lifestyle | | |
| Intervene in a multifactorial way including food, physical activity and behavior. Involve the family in the changes | 10 | AND, ICSI, INESSS, EnS, HAS, IHCW, NICE, NHMRC, NZMH, SIGN |
| Recommendations regarding intervention on physical activity | | |
| ≥ 60 minutes/day of moderate to vigorous intensity (games, sport, housework, activities of daily living, trips…), at least 10 minutes/session. | 10 | AAP, EASO, HAS, ICSI, IHCW, INESSS, NHMRC, NICE, SIGN, SIP |
| Physical activity enjoyed and chosen by the child | 8 | AAP, CMA, EASO, ICSI, INESSS, NICE, NZMH, SIGN |
| Promote a regular physical activity (walk the dog, walking, steps…) | 4 | EASO, NHMRC, NICE, SIGN |
| Promote physical activity as a family or with friends | 4 | EASO, ICSI, NHMRC, NZMH |
| Physical activity adapted to the child’s age, mental, and physical capabilities | 4 | CMA, INESSS, NICE, SIGN |
| Promote walking or cycling to school | 3 | EASO, ICSI, NZMH |
| Promote a structured/team sport (club) | 3 | EASO, NHMRC, NICE |
| Mix endurance and resistance exercises | 3 | CMA, INESSS, SIP |
| Parents should be role models | 2 | ICSI, NHMRC |
| ≥ 20 minutes/day of moderate to vigorous intensity, increasing gradually the time to reach a 60 minutes/day ideally | 1 | EnS |
| Identify the barriers to physical activity (time, fear, injury, economic, security) | 1 | ICSI |
| « Active » video games (not systematically recommended) | 1 | SIP |
| 0-3 years old:   - Supervised physical activity through active play | 1 | ICSI |
| 4-6 years old:   - 120 minutes/day of moderate to vigorous intensity - 60 minutes of structured activity, 60 minutes of non-structured activity - Walk to school | 1 | ICSI |
| 6-9 years old:   - ≥ 60 minutes/day of moderate to vigorous intensity - Predominatly aerobic activity (endurance) - Free play and organized sport (if the rules are few and flexible) - ≥ 3x/week: muscle and bone strengthening (running, jumping…) | 1 | ICSI |
| 10-12 years old:   - ≥ 60 minutes/day of moderate to vigorous intensity - Predominatly aerobic activity (endurance) - Emphasize physical activity as a family or with friends - ≥ 3x/week: muscle and bone strengthening (running, jumping…) | 1 | ICSI |
| Teenagers:   - ≥ 60 minutes/day of moderate to vigorous intensity - Predominatly aerobic activity (endurance) - Physical activity enjoyed by the teenager - Include friends - Include personal wellness activities (yoga, dance) in addition to a sport | 1 | ICSI |
| <5 years old:   - Indoor and outdoor active play/activities to develop skills - Family activities | 1 | NZMH |
| 5-18 years old:   - 5-10 minutes/day of aerobic activity, increasing time every week to reach 60 minutes/day ideally (taking the stairs, active play, houseworking, walking...) - ≥ 3x/week: muscle and bone strengthening (running, jumping…) | 1 | NZMH |
| Overweight children (1-2 years old):   - Offer opportunities to move | 1 | AAP |
| N1: Prevention Plus :   - ≥ 60 minutes/day of moderate to vigorous intensity (can be split in several sessions) - Unstructured active play and games are more suitable with youth - Sport has to be enjoyed by the child | 2 | AAP, ICSI |
| N2: Structured Weight Management :   - ≥ 60 minutes/day of supervised and planned moderate to vigorous intensity | 2 | AAP, ICSI |
| N3: Comprehensive multidisciplinary intervention :   - Behavior change plan including food monitoring, short-term goals for food and physical activity and contingency management | 2 | AAP, ICSI |
| Recommendations regarding intervention on sedentary behaviors | | |
| ≤ 2 hours/day of non-academic screen time | 8 | AAP, EASO, EnS, ICSI, IHCW, INESSS, NZMH, SIGN |
| Decrease screen time/physical inactivity (according to personal habits and environment) | 6 | AND, CMA, HAS, NHMRC, NICE, SIP |
| No television in the bedroom | 2 | AAP, EnS |
| < 14 hours/week of non-academic screen time | 1 | SIGN |
| 0-3 years old:   - No screens before 2 years old | 3 | HAS, ICSI, NZMH |
| Overweight children (1-2 years old):   - Limit screen time - No television in the bedroom | 1 | AAP |
| < 5 years old:   - Reduce sitting time without moving (for example: reduce car travel time) - From 2 years: 1 hour/day of screen time | 1 | NZMH |
| 4-6 years old:   - ≤ 2 hours/day of screen time | 1 | ICSI |
| N1: Prevention Plus   - < 2 years old : no screen - ≤ 2 hours/day of screen time - No television in the bedroom | 2 | AAP, ICSI |
| N2: Structured Weight Management :   - ≤ 1 hour/day of screen time | 2 | AAP, ICSI |
| Recommendations regarding intervention on behavior change and management | | |
| Stimulus control | 8 | AND, CMA, ICSI, IHCW, INESSS, NICE, NZMH, SIGN |
| Motivational interview | 8 | AAP, CMA, EASO, EnS, HAS, ICSI, IHCW, INESSS |
| SMART goals with child and/or parents, focused on the behavior/process more than on the weight change, adapted to age and context | 8 | AAP, CMA, ICSI, IHCW, INESSS, NHMRC, NZMH, SIGN |
| Behaviors/goals self-monitoring (notebook, weight, behavior, physical activity monitoring) | 7 | AAP, CMA, ICSI, IHCW, INESSS, NZMH, SIGN |
| Use non-food rewards | 5 | CMA, IHCW, INESSS, NZMH, SIGN |
| Problem-solving | 5 | CMA, IHCW, INESSS, NZMH, SIGN |
| Contingency management strategies | 5 | AAP, CMA, ICSI, INESSS, NZMH |
| Identify personal barriers | 4 | CMA, INESSS, NHMRC, NZMH |
| Self-management | 3 | CMA, ICSI, NZMH |
| Progressive, sustainable and little changes | 3 | CMA, NHMRC, SIGN |
| Positive reinforcement | 3 | CMA, HAS, NICE |
| Cognitive-behavioral therapy | 2 | AND, CMA |
| Relapse prevention | 1 | INESSS |
| Identify problematic behaviors/triggers | 1 | NZMH |
| Identify which intervention the child/parents want(s) to start with | 1 | NZMH |
| Fix an agreement with the goals | 1 | NZMH |
| Cognitive restructuring | 1 | NZMH |
| Social support | 1 | IHCW |
| Parents as models | 1 | NHMRC |
| Recommendations regarding who should be implicated in care coordination | | |
| Dietitian | 7 | AAP, AND, CMA, EASO, EnS, HAS, INESSS |
| Pediatrician | 6 | AAP, CMA, EASO, HAS, ICSI, INESSS |
| Physiotherapist | 4 | AAP, CMA, HAS, INESSS |
| Family physician | 4 | EASO, EnS, HAS, INESSS |
| Psychologist/child psychiatrist | 4 | CMA, EnS, HAS, INESSS |
| Exercice specialist | 3 | AAP, CMA, HAS |
| Nurse | 3 | AAP, EASO, INESSS |
| School health services | 2 | EASO, HAS |
| Social workers | 2 | HAS, INESSS |
| Psychoeducator | 1 | INESSS |
| Occupational therapist | 1 | INESSS |
| Pharmacist | 1 | INESSS |
| Sport’s physician | 1 | HAS |
| N1: Prevention Plus :   - Physicians - Specialized nurse | 1 | AAP |
| N2: Structured Weight Management :   - Dietitian - Clinician trained for meal plans - Team trained in the motivational interview and in positive reinforcement strategies - Family counselor - Physiotherapist/sports coach - Physician - Specialized nurse | 1 | AAP |
| N3: Comprehensive multidisciplinary intervention :   - Experienced multidisciplinary team | 1 | AAP |
| N4: Tertiary care intervention :   - Specialized center with interdisciplinary team experienced in child obesity and its comorbidities (including physician, nurse, psychologist, dietitian and physiotherapist) | 1 | AAP |
| Organization with interdisciplinary team | 3 | AND, CMA, INESSS |
| Separate interventions with interdisciplinary collaboration | 3 | AND, CMA, INESSS |
| Team trained on behavior and lifestyle change techniques and on therapeutic education | 3 | HAS, NICE, SIGN |
| Interventions with specialists in case of comorbidities or complex needs (for example: learning difficulties, eating disorder, no changes despite interventions, no impact on parents, family problems). Refer to the psychologist or dietitian. | 2 | NHMRC, NICE |
| Specialized organizations when there is a severe obesity with comorbidities or an endocrine cause to obesity | 1 | SIGN |
| Secondary :   - Ambulatory service with professionals trained in obesity | 1 | SIP |
| Tertiary :   - Specialized center for child obesity | 1 | SIP |
| Adapt the implication of professionals depending on the complexity of the situation with different professionals included depending on the levels | 1 | HAS |
| Recommendations regarding pharmacological interventions | | |
| Orlistat (low to moderate effectiveness)  Terms of use (variable according to guidelines):   - > 12 years old (teenagers) - With a lifestyle intervention - Obesity with comorbidities or cardiovascular risk factors or severe obesity - Monitoring of nutrition status and counseling by an experienced professional - Only if all the interventions of conservative treatment failed | 11 | AAP, AND, CMA, EnS, ICSI, INESSS, NHMRC, NICE, NZMH, SIGN, SIP |
| Do not use metformine | 2 | INESSS, NHMRC |
| Supplement with vitamin D | 1 | SIP |
| Supplement with micronutrients to ensure growth and development | 1 | NICE |
| Non-recommended for <12 years old children excepted when there are exceptional circumstances (severe comorbidities) | 1 | NICE |
| No treatment allowed | 1 | HAS |
| Interrupt treatment if there is no reduction of >4% in BMI or BMI z-score after 12 weeks | 1 | EnS |
| Try treatment for 6-12 months | 1 | NICE |
| N4: Tertiary care intervention :   - Sibutramine if >16 years old - Orlistat if >12 years old | 2 | AAP, ICSI |
| Recommendations regarding bariatric surgery | | |
| Indications for bariatric surgery |  |  |
| BMI > 40 or 35 with severe comorbidities (DM2, high blood pressure, non-alcoholic steatohepatitis, obstructive sleep apnea syndrome) | 6 | EnS, ICSI, NHMRC, NZMH, SIGN, SIP |
| Tanner’s stages at 4-5 or bone age ≥ 13 years old for girls and ≥ 15 years old for boys | 4 | EnS, ICSI, NZMH, SIGN |
| Failure of the weight management program >6 months | 5 | AND, EnS, ICSI, NZMH, SIP |
| Discernment ability and comprehension of the medical and dietetic implications | 4 | AND, EnS, ICSI, SIGN |
| Psychological assessment | 3 | AND, EnS, ICSI |
| Family support | 2 | AND, ICSI |
| Adherence to the principles of physical activity and balanced diet | 3 | AND, EnS, ICSI |
| Specialized center | 4 | AND, EnS, ICSI, NHMRC |
| Long-term follow-up | 3 | AND, EnS, ICSI |
| At least 15 years old (14 exceptionally) | 1 | NZMH |
| Contraindications for bariatric surgery |  |  |
| Preteen | 2 | EnS, ICSI |
| Non-management of the principles of physical activity and balanced diet | 2 | EnS, ICSI |
| Eating disorder, non-treated psychological problems | 3 | EnS, ICSI, NZMH |
| Prader-Willy syndrome (or another one) | 2 | ICSI, NZMH |
| Pregnancy, breastfeeding, pregnancy considered in the next 2 years | 4 | EnS, ICSI, NZMH, SIP |
| Alcohol dependence | 2 | EnS, SIP |
| Inability to take care of oneself | 1 | SIP |
| Lack of adherence to the treatment programs | 1 | SIP |
| Decreased life expectancy linked with illnesses | 1 | SIP |
| Chronic inflammatory bowel disease | 1 | SIP |
| Not recommended excepted in case of resistance to treatment, major complications and on the advice of a specialized team | 2 | CMA, HAS |
| Generally not recommended for children and teenagers. May be considered for teenagers only in exceptional circumstances or if they have (almost) reached the psychological maturity | 1 | NICE |
| N4: Tertiary care intervention :   - BMI ≥ 40 with a comorbidity or ≥ 50 - Physical, emotional and cognitive maturity (≥ 13 years old for girls, ≥ 15 years old for boys) - Weight loss try ≥ 6 months with treatment including behavioral change - Dietetic and psychological preoperative assessment and postoperative follow-up | 2 | AAP, ICSI |
| Recommendations regarding taking in account comorbidities in the nutritional intervention | | |
| To take into consideration comorbidities | 9 | AAP, EASO, EnS, HAS, ICSI, INESSS, NICE, SIGN, SIP |
| Manage comorbidities as soon as they have been identified (do not wait for the effect of weight loss) | 1 | NICE |
| Recommendations regarding intervention on other factors | | |
| Send to a psychologist in case of distress (low self-esteem, perceived as repulsive, depression, eating disorder, body dissatisfaction) | 1 | SIGN |
| Improve sleep :   - Regularity of duration - Regularity of bedtime - Comfortable environment - No distraction (screen) where children sleep - No caffeine   Recommended sleep hours :   - 0-3 months : 14-17 hours - 4-11 months : 12-15 hours - 1-2 years old : 11-14 hours - 3-4 years old : 10-13 hours - 5-13 years old : 9-11 hours - 14-17 years old : 8-10 hours - 18-25 years old : 7-9 hours | 1 | NZMH |
| Give child and family information about:   - Overweight and its implications on health - Realistic weight loss goals - Distinction between weight loss and maintenance and the importance of finding skills for both - Realistic goals for other outcomes (physical activity, food) - Healthy food - Personal care - Volunteer organizations and support groups (contact) | 1 | NICE |

MONITORING

| Item mentioned in guidelines | Number of guideline-s mentioning it | References of the guidelines |
| --- | --- | --- |
| Type of follow-up and monitoring | | |
| Individual or group follow-up (according to age, development stage and situation) | 2 | AND, SIP |
| Multidisciplinary follow-up that may include group sessions with parents, physical activity workshops and internships, short or extended (>2 months) stays in rehabilitation facilities | 1 | HAS |
| N2: Structured Weight Management :   - In some cases, group sessions are more efficient and effective | 1 | AAP |
| N3: Comprehensive multidisciplinary intervention :   - Group sessions are more economic and have a better therapeutic benefice | 1 | AAP |
| Duration of follow-up and monitoring | | |
| Long term follow-up | 4 | CMA, IHCW, NHMRC, NICE |
| ≥ 6 months | 3 | AND, INESSS, SIGN |
| Minimum 2 years | 1 | IHCW |
| Minimum 3 months when there is a diet prescription | 1 | CMA |
| 2 distinct phases:  Intensive phase :   - At least 3 months or when weight goals are achieved   Support phase :   - Frequent contact | 1 | AND |
| N1: Prevention Plus :   - 3-6 months - If there is no improvement, switch to protocol N2 | 2 | AAP, ICSI |
| N2: Structured Weight Management :   - 3-6 months - If there is no improvement, switch to protocol N3 | 2 | AAP, ICSI |
| N3: Comprehensive multidisciplinary intervention :   - Minimum 8-12 weeks | 1 | ICSI |
| Follow-up and monitoring frequency | | |
| Intensive frequency of follow-up | 1 | SIGN |
| Adapted to the child/teenager and his/her needs | 1 | INESSS |
| Frequent contact especially in the short term according to the child’s needs. Reduce the frequency when the goals are achieved. | 1 | NHMRC |
| Monthly to quarterly | 1 | HAS |
| At least once a month. If possible, once a week during the 3 first months | 1 | EnS |
| Close follow-up when there is a meal plan prescription | 1 | AND |
| N1: Prevention Plus   - Once a month - Suitable to the family - To use motivational interviewing to define frequency | 2 | AAP, ICSI |
| N2: Structured Weight Management :   - Once a month | 2 | AAP, ICSI |
| N3: Comprehensive multidisciplinary intervention :   - Once a week during 8-12 weeks - Once a month during the support phase | 2 | AAP, ICSI |
| Evaluation and monitoring indicators | | |
| Food : | 4 | AAP, ICSI, NHMRC, SIP |
| Intakes frequency, portions size, quality, drinks | 1 | NHMRC |
| Eating disorder (Binge-eating disorder, Night-eating syndrome) and food intake without hunger | 2 | NHMRC, SIP |
| Family approach (supplies responsibility, meals choice and preparation, involvement, meals location, outside meals) | 1 | NHMRC |
| Parental approach (model, use of food as a reward) | 1 | NHMRC |
| Behavior related to food | 1 | SIP |
| Physical activity : | 4 | AAP, ICSI, NHMRC, SIP |
| Physical inactivity/day (transports, screens) | 2 | NHMRC, SIP |
| Planned or unplanned activity (active time, outside, structured exercise) | 1 | NHMRC |
| Physical and sedentary activities as a family | 1 | NHMRC |
| Parental model | 1 | NHMRC |
| Anthropometric measurements : | 10 | AAP, AND, EnS, HAS, ICSI, IHCW, NHMRC, NZMH, SIGN, SIP |
| BMI | 2 | EnS, HAS |
| BMI (every 3 to 6 months) | 2 | EnS, NHMRC |
| BMI (goal: < 85th percentile or -0.5 au z-score) | 1 | SIP |
| BMI z-score (goal: 0.5 in 0 to 6 months and ≥ 0.6 in 6 to 12 months) | 1 | AND |
| BMI (once a year) | 1 | SIGN |
| Weight (every 3 to 6months) | 2 | NHMRC, NZMH |
| Height (every 3 to 6months) | 2 | NHMRC, NZMH |
| Waist size | 2 | HAS, NHMRC |
| Maintaining weight loss | 1 | IHCW |
| Changes made according to the goals | 1 | HAS |
| Encountered difficulties (to fix new goals if necessary) | 1 | HAS |
| Terms of support and pace of follow-up | 1 | HAS |
| Psychosocial factors   - Weight control behavior (exercise, food restriction, products to lose weight, vomiting/laxatives, related parental speech and model) - Family’s perception of the body image (body perception, body checks and avoidant behaviors, thoughts and beliefs related to the body, distress related to weight, related parental speech and model) - Psychosocial factors (harassment, mocking…) - Familial functioning and ability to make behavioral changes | 1 | NHMRC |
| Physical appareance | 1 | SIP |
| Life quality | 1 | SIP |
| In the case of an energy restriction:   - Micronutrient intake - Height and weight growth | 1 | AND |
| N2: Structured Weight Management :   - Notebook on 3 days (food, screen time, physical activity) | 1 | AAP |
| N3: Comprehensive multidisciplinary intervention :   - Systematic assessment of the anthropometric measurements - Food and physical activity assessment at regular intervals | 1 | AAP |
| Recommendations regarding kinetic of weight and/or weight loss | | |
| Overweight and obesity during growth (severe obesity excluded)   - Weight maintenance | 7 | AND, EASO, EnS, HAS, ICSI, NHMRC, NICE |
| Overweight and obesity during growth (severe obesity excluded):   - Low weight loss can stem from lifestyle changes | 1 | EASO |
| Severe obesity :   - Maximum loss of 0.5-1 kg/month | 1 | ICSI |
| Severe obesity :   - 7% loss of initial weight | 1 | EnS |
| Teenagers at the end of growth:   - In a limited time, loss of 1-2kg/month | 1 | EASO |
| Teenagers at the end of growth:   - Weight stabilization or very gradual loss | 1 | HAS |
| Teenagers at the end of growth:   - Weight loss | 1 | NHMRC |
| > 11 years old:   - Overweight: weight maintenance - Obesity: maximum 1kg/week loss | 1 | SIP |
| 6-11 years old:  Obesity   - Weight maintenance until reaching the 80^th^ percentile BMI or - Maximum 0.5kg/month loss   Severe obesity   - Maximum 1kg/week loss | 1 | INESS |
| 12-18 years old:  Obesity   - Weight maintenance until reaching the 85^th^ percentile BMI or - Maximum 1kg/month loss   Severe obesity   - Maximum 1kg/week loss | 1 | INESS |
| N1: Prevention Plus :   - Weight maintenance - BMI reduction | 2 | AAP, ICSI |
| N2: Structured Weight Management :   - Weight maintenance - 2-11 years old: maximum 0.5kg/month loss - 12-18 years old: maximum 1kg/week loss | 2 | AAP, ICSI |
| N3: Comprehensive multidisciplinary intervention :   - Weight maintenance - Weight maintenance until reaching the 85^th^ percentile BMI - 2-5 years old: maximum 0.5kg/month loss - 6-18 years old: maximum 1kg/month loss | 1 | AAP |
| Do not systematically set weight goal. If a weight goal is set, assess the relevance of communicating that goal to the child/family (according to context) | 1 | INESSS |
| Investigate patient’s goal | 1 | HAS |
| Induce weight loss if comorbidities are present | 2 | HAS, SIGN |

Full description and references of the 17 guidelines :

AAP American Academy of Pediatrics [1]

AND Academy of Nutrition and Dietetics [2-4]

CMA Canadian Medical Association [5]

EASO European Association for the Study of Obesity [6]

EnS Endocrine Society [7]

HAS Haute Autorité de Santé [8]

ICSI Institute for Clinical Systems Improvement [9]

IHCW Institute for Healthy Childhood Weight [10]

INESSS Institut national d’excellence en santé et en service sociaux [11]

NHMRC National Health and Medical Research Council [12]

NICE National Institute for Health and Care Excellence [13]

NZMH New Zealand Ministry of Health [14,15]

SIGN Scottish Intercollegiate Guidelines Network [16]

SIP Società Italiana di Pediatria [17]

1. Spear, B.A.; Barlow, S.E.; Ervin, C.; Ludwig, D.S.; Saelens, B.E.; Schetzina, K.E.*, et al.* Recommendations for Treatment of Child and Adolescent Overweight and Obesity. *Pediatrics* **2007**, *120*, S254-S288.

2. Academy of Nutrition and Dietetics. Pediatric Weight Management. Major recommendations. <https://www.andeal.org/vault/pq57.pdf> (10.12.2018)

3. Academy of Nutrition and Dietetics. Pediatric Weight Management. Major recommendations. <https://www.andeal.org/vault/pq140.pdf> (10.12.2018)

4. Hoelscher, D.M.; Kirk, S.; Ritchie, L.; Cunningham-Sabo, L. Position of the Academy of Nutrition and Dietetics: Interventions for the Prevention and Treatment of Pediatric Overweight and Obesity. *Journal of the Academy of Nutrition and Dietetics* **2013**, *113*, 1375-1394.

5. Lau, D.C.; Douketis, J.D.; Morrison, K.M.; Hramiak, I.M.; Sharma, A.M.; Ur, E.*, et al.* 2006 Canadian clinical practice guidelines on the management and prevention of obesity in adults and children [summary]. *CMAJ* **2007**, *176*, S1-13.

6. Baker, J.L.; Farpour-Lambert, N.J.; Nowicka, P.; Pietrobelli, A.; Weiss, R.; Childhood Obesity Task Force of the European Association for the Study of Obesity. Evaluation of the overweight/obese child--practical tips for the primary health care provider: recommendations from the Childhood Obesity Task Force of the European Association for the Study of Obesity. *Obesity Facts* **2010**, *3*, 131-137.

7. Styne, D.M.; Arslanian, S.A.; Connor, E.L.; Farooqi, I.S.; Murad, M.H.; Silverstein, J.H.*, et al.* Pediatric Obesity-Assessment, Treatment, and Prevention: An Endocrine Society Clinical Practice Guideline. *J Clin Endocrinol Metab* **2017**, *102*, 709-757.

8. Haute Autorité de Santé. Surpoids et obésité de l'enfant et de l'adolescent. <https://www.has-sante.fr/portail/jcms/c_964941/fr/surpoids-et-obesite-de-l-enfant-et-de-l-adolescent-actualisation-des-recommandations-2003> (22.12.2018),

9. Institute for Clinical Systems Improvement. *Prevention and Management of Obesity for Children and Adolescents*; 2013.

10. Altman, M.; Wilfley, D.E. Evidence update on the treatment of overweight and obesity in children and adolescents. *Journal of Clinical Child and Adolescent Psychology: The Official Journal for the Society of Clinical Child and Adolescent Psychology, American Psychological Association, Division 53* **2015**, *44*, 521-537.

11. Institut national d'excellence en santé et en services sociaux. *Traitement de l'obésité des enfants et des adolescents en 1re et 2e ligne: guide de pratique clinique. Volet I*; INESSS: Montreal, Québec, 2012.

12. National Health Medical Research Council. *Clinical practice guidelines for the management of overweight and obesity in adults, adolescents and children in Australia*; National Health and Medical Research Council: Melbourne, 2013.

13. National Clinical Guideline Centre. *Obesity: Identification, Assessment and Management of Overweight and Obesity in Children, Young People and Adults: Partial Update of CG43*. National Institute for Health and Care Excellence (UK): London, 2014.

14. Ministry of Health. *Clinical Guidelines for Weight Mangagement in New Zealand Children and Young People*; Ministry of Health: Wellington, 2016.

15. Ministry of Health; Clinical Trials Research Unit. *Clinical Guidelines for Weight Management in New Zealand Children and Young People*; Ministry of Health: Wellington, 2009.

16. Scottish Intercollegiate Guidelines Network. *Management of obesity: a national clinical guideline*. Scottish Intercollegiate Guidelines Network: Edinburgh, 2010.

17. Società Italiana di Pediatria; Società Italiana di Endocrinologia e Diabetologia Pediatrica. Consensus su diagnosi, trattamento e prevenzione dell'obesita del'bambino e dell'adolescente. <https://docs.sip.it/Consensus_Obesita_2017.pdf> (22.12.2018),

1. Food classified in three colors according to it’s nutritional quality : green (low energy-density and high nutritional quality food, to be frequently consumed), yellow (moderate energy-density food (cereals), to be consumed moderately), red (high energy-density and low nutritional quality food, to be consumed occasionally) (139–141) [↑](#footnote-ref-1)
